# Graphene-Supported Thin Metal Films for Nanophotonics and Optoelectronics

**DOI:** 10.3390/nano8121058

**Published:** 2018-12-15

**Authors:** Dmitry I. Yakubovsky, Yury V. Stebunov, Roman V. Kirtaev, Kirill V. Voronin, Artem A. Voronov, Aleksey V. Arsenin, Valentyn S. Volkov

**Affiliations:** 1 Center for Photonics & 2D Materials, Moscow Institute of Physics and Technology, 9 Institutsky Lane, Dolgoprudny 141700, Russia; dmitrii.yakubovskii@phystech.edu (D.I.Y.); stebunov@phystech.edu (Y.V.S.); rkirtaev@gmail.com (R.V.K.); voronin.kv@phystech.edu (K.V.V.); voronov.artem@gmail.com (A.A.V.); arsenin.av@mipt.ru (A.V.A.); 2 GrapheneTek, 7 Nobel Street, Skolkovo Innovation Center, Moscow 143026, Russia; 3 SDU Nano Optics, Mads Clausen Institute, University of Southern Denmark, Campusvej 55, DK-5230 Odense, Denmark

**Keywords:** graphene, thin metal films, graphene/metal hybrid nanostructures, optical constants, gold, copper, spectroscopic ellipsometry

## Abstract

Graphene-metal hybrid nanostructures have attracted considerable attention due to their potential applications in nanophotonics and optoelectronics. The output characteristics of devices based on such nanostructures largely depend on the properties of the metals. Here, we study the optical, electrical and structural properties of continuous thin gold and copper films grown by electron beam evaporation on monolayer graphene transferred onto silicon dioxide substrates. We find that the presence of graphene has a significant effect on optical losses and electrical resistance, both for thin gold and copper films. Furthermore, the growth kinetics of gold and copper films vary greatly; in particular, we found here a significant dependence of the properties of thin copper films on the deposition rate, unlike gold films. Our work provides new data on the optical properties of gold and copper, which should be considered in modeling and designing devices with graphene-metal nanolayers.

## 1. Introduction

In recent years, a great deal of interest has been directed toward the use of graphene/metal hybrid nanostructures in the development of high-efficiency photonic, plasmonic, optoelectronic and nanoscale electronic devices [1,2,3]. Graphene is an atomically thin layer which therefore in practice does not affect the size of nanostructures, while possessing many remarkable properties, including broadband optical transparency [4,5,6], outstanding transport (electrical and thermal conductivity) [7,8,9,10] and record-breaking strength [11]. In addition, monolayer graphene is impermeable to atoms, molecules, ions, liquids, and standard gases [12,13,14] and exhibits superior chemical and thermal stability [15,16] together with high bioreactivity and biocompatibility [17,18]. Notably, the combination of graphene and other materials, in most cases, positively affects the entire hybrid structure. Thus, one of the most exciting characteristics of metal/graphene (plasmonic) hybrid devices is the tunability of their optical properties, which paves the way for new applications in the visible-near-IR spectral region [19,20,21,22,23,24]. Moreover, metal/graphene hybrid structures allow one to obtain a significant improvement in the strength and fatigue limit in bending compared to conventional metal structures without graphene [25,26], which allows an increase in the functionality of thin-film metal structures in relation to their use in flexible electronics applications.

Nanometric composite hybrid structures consisting of alternating thin layers of metal deposited onto a sublayer of graphene have been used to demonstrate room temperature operation of novel ultrasensitive broadband photodetectors [21,24,27], photovoltaic solar cells [19,28], hybrid graphene/metasurface systems at terahertz and infrared frequencies [24,29,30,31,32] and highly sensitive surface-enhanced Raman scattering (SERS) biosensors with the fluorescence quenching effect [33,34,35]. In addition, such novel composites can be potentially used as transparent graphene-based neural interfaces for electrophysiology, in vivo neural imaging, optogenetic applications [36,37] and brain-computer interfaces [38]. Furthermore, the combination of metallic components with other 2D materials to form a hybrid nanostructure system also provides a multifunctional platform with enhanced performance for various applications [39,40,41].

The fabrication of hybrid structures can be accomplished by transferring graphene (or a metal) to a metal (or graphene) by using a transferring method [20,42] or by the direct deposition of metals on single-layer graphene (for example by physical vapor deposition); in practice, both methods are used. In the first case, the optical, electrical and structural properties of a thin metal film cannot differ significantly from the standard tabulated values [43,44], and to our knowledge, no experimental studies have been done to clarify the second case: The effects of the graphene sublayer on the properties of the deposited metal. At present, the initial growth kinetics of nanoparticles of various metals on the surface of graphene (for both chemical vapour deposited (CVD) and mechanically exfoliated samples) [45,46] has been studied in some detail, but little attention has been paid to the deposition of thin metallic films on graphene and the study of their properties. One notable exception is the recent work of Majkova et al. [47], where the growth kinetics of copper films on CVD graphene transferred onto an oxidized silicon surface was studied in detail using the grazing-incidence small-angle X-ray scattering (GISAXS) technique, but the optical properties of these films as well as their conductivities have not been investigated. It has been noted that the ratio of the adsorption energy of copper adatoms on graphene to the bulk cohesive energy of the copper is sufficiently small, causing the graphene substrates to become non-wettable by copper. In particular, the latter circumstance (in combination with the very small diffusion barrier for copper on graphene) results in copper growing on the surface of graphene in the form of 3D islands during the initial stages according to the well-known Volmer–Weber model of thin film growth [48,49]. In addition, a 15 nm-thick copper film on graphene is close to the percolation threshold, whereas continuous films of the same thickness can be obtained for silicon, optical glass or other typical optical substrates [50].

In the present work, we have investigated the influence of a graphene underlayer on Au and Cu films properties in continuous thin films at thicknesses of 25 and 50 nm. Gold was chosen because, to date, it has been the most popular material for a variety of applications in nanoplasmonics. The optical properties of thin polycrystalline copper films are not only as good as those of noble metals [51,52,53], but unlike the noble metal components, copper-based components can easily be implemented in integrated circuits using industry-standard fabrication processes. It has been previously shown that the properties of thin-film plasmonic nanostructures depend appreciably on the film thickness [44,54,55,56], deposition rate [51,57] and ambient temperature [58]. Accurate accounting for optical constants is important for theoretical and numerical analyses (modeling) of thin-film hybrid graphene-metal nanostructures. We have undertaken numerous experiments with different deposition regimes to study the change in the optical and electrical properties of Cu and Au films with respect to differences in structural features. We focused on observing how the graphene underlayer affects the growth kinetics and characteristics of continuous thin films. All deposited films were characterized using the following techniques: Scanning electron microscopy (SEM), atomic force microscopy (AFM), and X-ray diffraction (XRD) to study the structural morphology; four-point probe measurements to determine the electrical properties; and spectroscopic ellipsometry to determine the optical constants in the spectral range from 300 to 3300 nm. The results of the experimental study are demonstrated in detail below.

## 2. Experimental Section

### 2.1. Sample Fabrication

As substrates for thin metal films deposition, we used CVD-grown monolayer graphene transferred by a poly(methyl methacrylate) (PMMA)-assisted transfer method onto silicon wafers with a 285 nm SiO_2_ coating (from Graphene Laboratories Inc. (New York, NY, USA)). Monolayer graphene covered more than 90% of the substrate area, and the remaining part of the substrate was filled with a random distribution of bilayer graphene islands. Before metal deposition, graphene substrates were annealed at 250 °C in a vacuum chamber 10^−6^ Torr for 1 h to remove residual PMMA and water.

The crystal quality of monolayer graphene was assessed with a Thermo Scientific™ DXR™2 Raman microscope equipped with a 532 nm excitation laser working at a power of 10 mW. Raman spectra were acquired through a 50× microscope objective by means of 10 consecutive exposures, each with a duration time of 15 s. The ratio of the intensities of the D and G peaks located near 1340 and 1580 cm^−1^, respectively, indicates the defect level in the graphene crystal [59].

Deposition of metals was made both on the graphene/SiO_2_/Si and similar SiO_2_/Si wafers without graphene to compare the properties of films on different substrates.

All film depositions were performed using gold and copper pellets with a purity of 99.999% (Kurt J. Lesker), and the e-beam evaporation procedure employing a Nano-Master NEE-4000 system. The base pressure of a vacuum chamber before and during the evaporation process was ~7 × 10^−7^ Torr and ~4 × 10^−6^ Torr, correspondingly. Unless mentioned otherwise, all metal depositions were performed at 20 °C. The nominal thicknesses of the deposited films are approximately 25 and 50 nm with a deposition rate in the range from 1 to 15 Å/s and were monitored by a quartz-crystal mass-thickness sensor (inside the evaporator).

### 2.2. Structural Characterization

X-ray diffraction (XRD) measurements of the (111) peaks of Au and Cu (Thermo ARL X’TRA X-ray diffractometer with Cu K_α_ radiation (*λ* = 0.154 nm)) were used to investigate the crystallinity of the deposited films. The Bragg reflection peak was analyzed by the Debye-Scherrer formula $\langle D\rangle =\frac{\lambda }{\sqrt{\beta^{2}-\beta_{\mathrm{std}}^{2}}\cos\theta }$ to estimate the average size of crystallites using the values of the peak width β, where θ is the diffraction peak angle and $\beta_{\mathrm{std}}=0.131$ is the instrumental broadening determined by the corundum single-crystal standard (SRM NIST).

Surface morphology was studied by scanning electron microscopy (SEM) (JEOL JSM-7001F with a Schottky emitter in secondary electron imaging mode, with a voltage of 30 kV, and a working distance of ~6.2–6.4 mm).

Topography measurements were performed using an atomic force microscopy (AFM, NT-MDT Ntegra) in tapping mode with a probe resonance frequency of 250 kHz. To determine the root-mean-square surface roughness of thin films, an area of 1.5 × 1.5 μm was used. The thickness of each film was obtained by direct measurement of the step height by an AFM scan of the film surface.

### 2.3. Optical and Electrical Characterization

To investigate the optical characteristics of thin metal films we applied ellipsometry measurements using a variable-angle spectroscopic ellipsometer VASE^®^ by J. A. Woollam Co. (Lincoln, NE, USA) in the wavelengths range of 300–3300 nm. Ellipsometry parameters Ψ and Δ were measured at angles of incidence of 70° and 75°, and to extract the complex effective dielectric function of metal films, ellipsometry data were analyzed by the Drude-Lorentz model fitting applying specialized spectroscopic ellipsometry software (WVASE^®^, J. A. Woollam Co., Lincoln, NE, USA).

The sheet resistance of the thin metal films was measured by the four-point probe method on a Jandel RM3000 probe station using a head with a pin distance and radius of approximately 425 and 40 μm, correspondingly, both being much smaller than the area of deposited films (~1 × 1 cm^2^).

## 3. Results and Discussion

**Figure 1:**
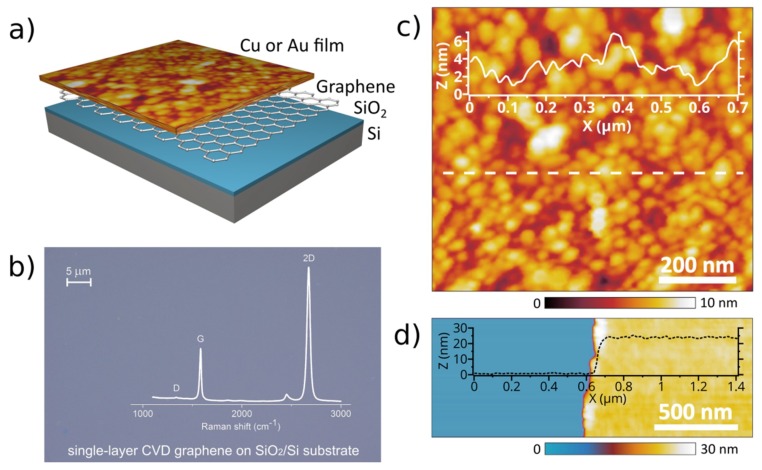
(**a**) Illustration of the sample structure. (**b**) An optical microscopy image and Raman spectrum of single-layer graphene transferred onto a SiO_2_/Si substrate. (**c**) Atomic force microscopy (AFM) surface morphology images of the deposited copper film (copper crystallites formed during the deposition). (**d**) The AFM image of the scratch on a thin copper film deposited on the graphene underlayer of a SiO_2_/Si substrate and the height profile of the scratched copper film.

**Figure A1:**
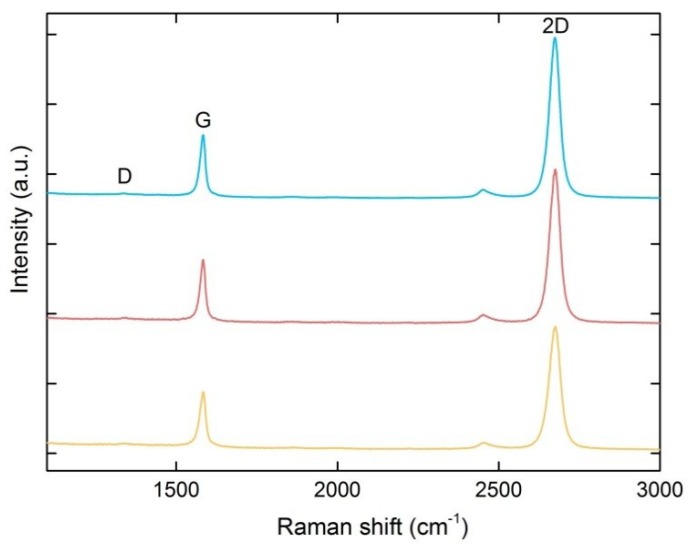
Raman spectra of single-layer graphene transferred onto a SiO_2_/Si substrate measured at three different sites. The near-absence of D peak demonstrates a low defect density of the crystal structure of graphene.

The growth and properties of thin metal films depend strongly on substrate characteristics and the use of additional coatings. In this study, we investigated metal films with thicknesses of several tens of nanometers deposited by electron beam evaporation on SiO_2_/Si wafers with and without a top graphene layer (Figure 1a). Raman spectra measured at several sites on the graphene surface indicate the near-absence of D peak and, therefore, a low defect density of its crystal structure (Figure 1b and Figure A1). A quartz crystal microbalance (QCM) installed in the e-beam system provides a means to control the thickness of metal films during deposition, while the exact thicknesses were assessed with AFM measurements for each sample. Figure 1c,d shows, respectively, a polycrystalline structure of the film and typical AFM images of a scratch made on the metal film. Accurate thickness measurements of metal films are of high importance in determining their optical properties with ellipsometric measurements [44]. For convenience, in this work, we used thickness values obtained with the QCM. AFM measurements of the grain structures of all metal films provided a root-mean-square surface roughness with amplitudes of less than 1.6 nm and 0.7 nm for the SiO_2_/Si substrates with and without top graphene layers, respectively.

### 3.1. Thin Gold Films

**Figure 2:**
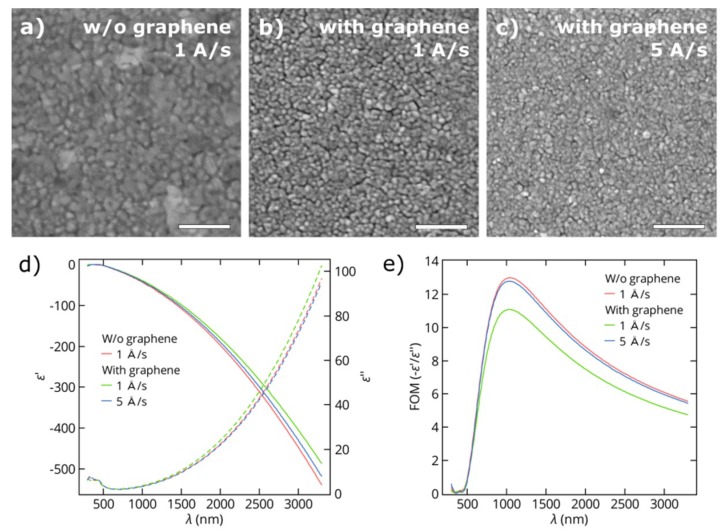
Scanning electron microscopy (SEM) micrographs (scale bars = 200 nm) of thin gold films (25 nm thick) deposited on (**a**) a SiO_2_/Si substrate at a rate of 1 Å/s, (**b**) a graphene/SiO_2_/Si substrate at a rate of 1 Å/s and (**c**) a graphene/SiO_2_/Si substrate at a rate of 5 Å/s. (**d**) The measured real $\varepsilon^{\prime }$ (solid line) and imaginary $\varepsilon^{\prime \prime }$ (dashed line) parts of the dielectric functions of Au films on SiO_2_/Si substrates with and without a graphene underlayer. (**e**) Figures of merit $-\varepsilon^{\prime }/\varepsilon^{\prime \prime }$ for the same Au films.

**Figure A2:**
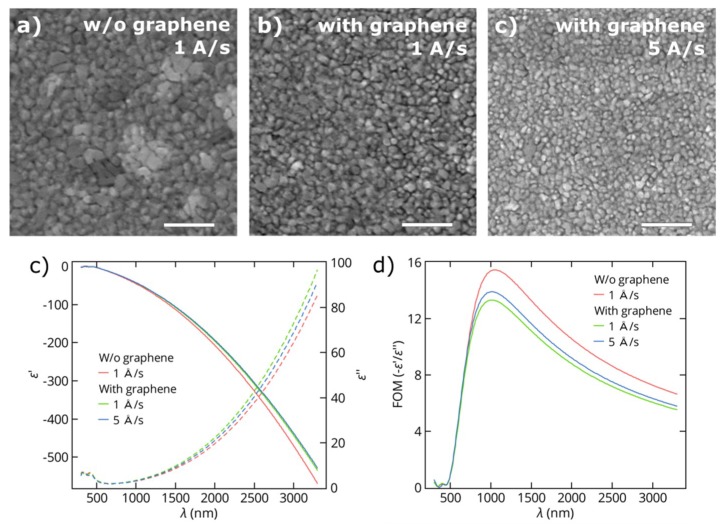
SEM micrographs (scale bars = 200 nm) of thin gold films (50 nm thick) deposited on (**a**) a SiO_2_/Si substrate at a rate of 1 Å/s, (**b**) a graphene/SiO_2_/Si substrate at a rate of 1 Å/s and (**c**) a graphene/SiO_2_/Si substrate at a rate of 5 Å/s. (**d**) The measured real *ε*′ and imaginary *ε*″ parts of the dielectric functions of Au films on SiO_2_/Si substrates with and without a graphene underlayer. (**e**) Figures of merit −*ε*′/*ε*″ for the same Au films.

To study the influence of graphene on the growth of metal films, thin gold films were deposited by electron beam evaporation onto SiO_2_/Si substrates with and without top graphene layers. In this section, we present the results for gold films with thicknesses of 25 nm (the results for 50 nm-thick gold films are shown in the Appendix A (Figure A2)). Figure 2a,b shows SEM images of Au films grown on SiO_2_/Si and graphene/SiO_2_/Si substrates at a rate of 1 Å/s in a single deposition process. These images reflect the formation of continuous polycrystalline metal films on both substrates and smaller average sizes of the in-plane grains of the gold films deposited on monolayer graphene. Gold films with the same thickness, but deposited on graphene at a higher rate of 5 Å/s, also consist of grains with smaller sizes comparing to gold films deposited on pure SiO_2_/Si substrates at the same rate (Figure 2c). This indicates different kinetics of gold growth on graphene and SiO_2_/Si substrates. XRD analysis of gold films reveals larger crystallite size for the films on SiO_2_ than for those on graphene. It further found that the size of gold crystallites on graphene also increases as the deposition rate goes up. Thus, we obtained a mean crystallite size of 23.9 nm for the films grown on SiO_2_ at both rates of 1 and 5 Å/s and mean crystallite sizes of 22.3 and 23.5 nm for the gold films on graphene deposited at 1 and 5 Å/s, respectively. A reduction of the mean crystallite size in the film results in an increase of the electron scattering losses at the crystallite boundaries, which leads to higher resistivity and optical losses. For this reason, in plasmonic and optoelectronic applications, the growth of gold films on graphene is preferred at higher deposition rates.

Next, to determine the effective permittivities of the gold films, we performed ellipsometric measurements and fitted the acquired data with a model comprising film thicknesses measured by AFM (Figure 2d). Figure 2e presents the figure of merit (FOM) for the optical quality of obtained gold films, which is the ratio of the real $\varepsilon^{\prime }$ and imaginary $\varepsilon^{\prime \prime }$ parts of the dielectric permittivity. For wavelengths higher than 1000 nm, the FOM for the gold films deposited on pure SiO_2_ is slightly higher than the FOM for the gold film deposited at the same rate on graphene with the overall difference less than 15%, which is mostly a consequence of the smaller crystallite size. This is true for deposition rates of both 1 and 5 Å/s. In addition, we performed sheet resistance measurements to characterize the electrical properties of deposited gold films. The sheet resistance of the gold films deposited on SiO_2_/Si substrates does not depend on the deposition rate and equals 2.0 Ohm/sq, which is 25% and 15% lower than the sheet resistance of the corresponding films deposited on graphene at the rates of 1 and 5 Å/s, respectively (2.7 Ohm/sq and 2.3 Ohm/sq). The data obtained for the optical FOM and sheet resistance of gold films are in a good agreement with the results of XRD analysis.

### 3.2. Thin Copper Films

**Figure 3:**
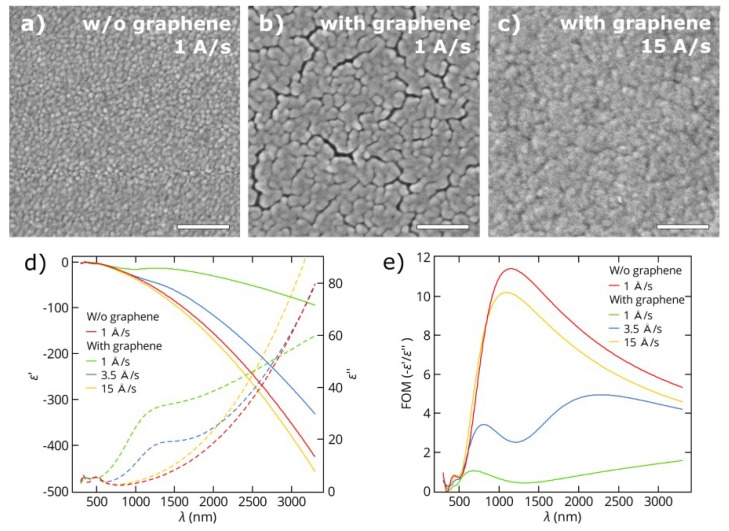
SEM micrographs (scale bars = 200 nm) of thin copper films (25 nm thick) deposited on (**a**) a SiO_2_/Si substrate at a rate of 1 Å/s, (**b**) a graphene/SiO_2_/Si substrate at a rate of 1 Å/s and (**c**) a graphene/SiO_2_/Si substrate at a rate of 15 Å/s. (**d**) The measured real $\varepsilon^{\prime }$ (solid line) and imaginary $\varepsilon^{\prime \prime }$ (dashed line) parts of the dielectric functions of Cu films on SiO_2_/Si substrates with and without graphene underlayer. (**e**) Figures of merit $-\varepsilon^{\prime }/\varepsilon^{\prime \prime }$ for the same Cu films.

In the same manner, we studied thin copper films deposited on SiO_2_/Si substrates with and without a top graphene layer. The results of the structural morphology and optical properties of copper films with a thickness of 25 nm are presented below. Additional results of 50 nm-thick copper films are given in the Appendix A. Figure 3a,b demonstrates the noticeable difference in morphology of 25 nm-thick copper films deposited at a low rate (approximately 1 Å/s) on SiO_2_/Si and graphene/SiO_2_/Si substrates in a single process. The copper films on a bare SiO_2_/Si substrate have a continuous structure, while the ones deposited on graphene contain narrow voids between metal clusters covering the entire film. Therefore, the addition of only a single atomic layer of graphene completely changes the growth kinetics of copper films.

**Figure A3:**
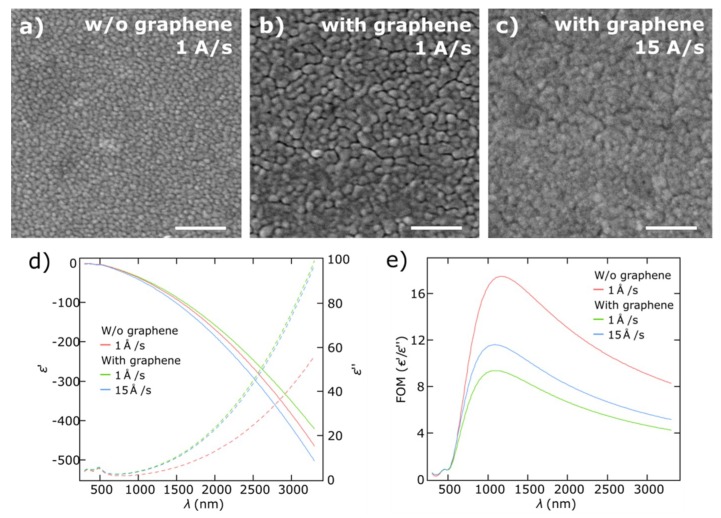
SEM micrographs (scale bars = 200 nm) of thin copper films (50 nm thick) deposited on (**a**) a SiO_2_/Si substrate at a rate of 1 Å/s, (**b**) a graphene/SiO_2_/Si substrate at a rate of 1 Å/s and (**c**) a graphene/SiO_2_/Si substrate at a rate of 15 Å/s. (**d**) The measured real *ε*′ and imaginary *ε*″ parts of the dielectric functions of Cu films on SiO_2_/Si substrates with and without a graphene underlayer. (**e**) Figures of merit −*ε*′/*ε*″ for the same Cu films.

Nevertheless, the deposition of continuous copper films on graphene is possible by increasing a number of nucleation centers during the initial steps of film growth. According to a report by Zaporojtchenko et al. [60] and a recent report by Schwartzkopf et al. [61] increasing the deposition rate by an order of magnitude leads a two-fold increase in the number of clusters formed. Based on this assumption, we carried out a series of depositions and investigated the dependence of the optical, electrical and structural characteristics of thin copper films on the deposition rate. As a result, we found that 25 nm-thick copper films deposited on graphene at higher deposition rates of 10–15 Å/s have a continuous surface morphology without notable voids (Figure 3с). The observed difference in morphology of the Cu films shown in Figure 3a–c is also reflected in their effective dielectric functions measured by spectroscopic ellipsometry. Figure 3d,e shows the real $\varepsilon^{\prime }$ and imaginary $\varepsilon^{\prime \prime }$ parts of dielectric functions and the FOM for copper films deposited on bare SiO_2_/Si at a rate of 1 Å/s and on graphene at rates of 1 to 15 Å/s. These results reveal the large discrepancy in the optical properties of the copper films deposited under the same conditions on SiO_2_/Si substrates with and without graphene, which showed poorer optical quality of the films with graphene due to presence of voids in their structure. However, an increase in the rate of copper deposition on graphene leads not only to the growth of more continuous films but also to gradual improvement in the optical FOM characterizing these films. Therefore, at a rate of 15 Å/s, the corresponding values of the FOM for copper films on graphene are quite close to those for films deposited on pure SiO_2_/Si substrates. Similar dependencies were obtained for copper films with a thickness of 50 nm (Figure A3). In addition, in Table A1 and Table A2 in the Appendix A, we summarize the dielectric constants of thin metal films, both Cu and Au, as well as their refractive indices *n* and extinction coefficients *k* acquired from ellipsometry measurements in a wavelength range of 300–3300 nm with a step of 10 nm.

**Figure 4:**
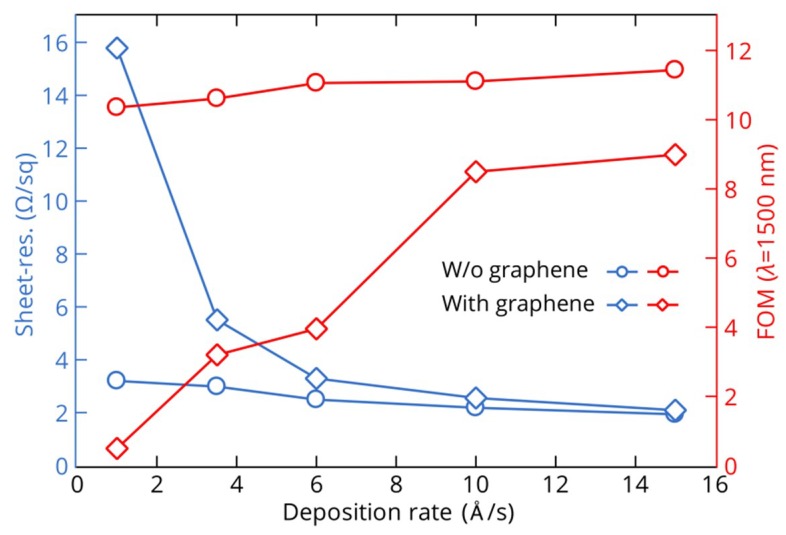
Dependences of sheet resistance and figure of merit (FOM) (*λ* = 1500 nm) on the deposition rate of thin copper films (25 nm thick) deposited on SiO_2_/Si substrates with (diamonds) and without (circles) graphene.

**Figure 5:**
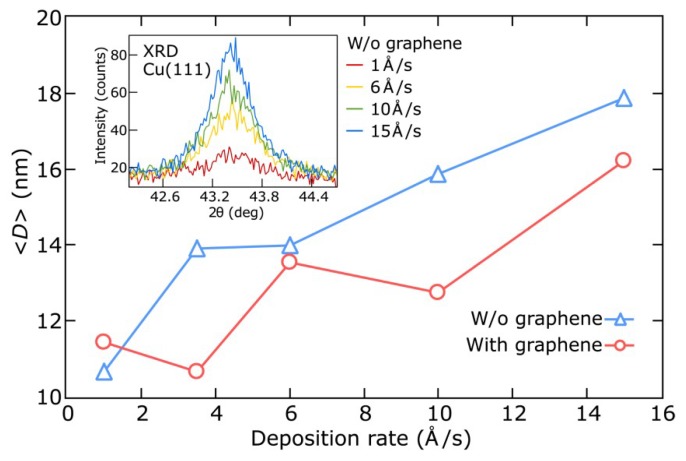
Dependences of the average crystallite size *D* (using X-ray diffraction (XRD) data) on the deposition rate ranging from 1 to 15 Å/s for thin copper films (25 nm thick) deposited on SiO_2_/Si substrates with (diamonds) and without (circles) graphene. Intensity enhancement of the XRD peak on the inset illustrates the improved crystallinity of the 25 nm-thick copper films deposited on SiO_2_/Si substrates without graphene.

Here, we analyze in more detail the dependence of electrical and optical properties of copper films as well as their crystalline structure on the film deposition rate. Figure 4 indicates the sheet resistance and FOM (at 1500 nm) of the 25 nm-thick copper films grown on SiO_2_/Si substrates with and without graphene at deposition rates ranging from 1 to 15 Å/s. Copper films deposited on graphene demonstrate noticeable improvements in sheet resistance as well as FOM for higher deposition rates (7.5- and 18-fold, respectively), while measurements of films on pure SiO_2_/Si substrates show only slight changes. Let us keep in mind that the deposition process determines the effective dielectric function of the film in two different ways: Influencing the crystallinity of the metal films and inducing the formation of voids in the film morphology. To analyze these impacts on the optical properties of the deposited films, we performed XRD measurements to determine the crystallinity of the films, where the average sizes of the crystallites were determined from the width of the XRD peaks using the Debye-Scherrer formula. Figure 5 shows an increase in the average crystallite size D with deposition rate for copper films with and without graphene. These results are consistent with those obtained in previous works for Au films on Si [62,63]. However, the crystallite sizes show the same dependencies on the deposition rate for both types of copper films, and therefore, the sheet resistance and FOM of copper films deposited on graphene at low rates are primarily defined by the presence of voids in percolated films. These film parameters become comparable with those of the films on SiO_2_/Si substrates only at high deposition rates when a more continuous film structure is formed. In addition, an increase in the crystallite sizes for higher deposition rates explains the corresponding improvement of 40% and 10% in electrical conductivities and optical response, respectively, of copper films on pure SiO_2_/Si substrates because of lower electron scattering at the crystallite boundaries.

### 3.3. Growth Mechanisms of Thin Metal Films on Graphene

Kinetics mechanisms of metal growth strongly influence both optical and electrical properties of metal films deposited on graphene. The formation of nuclei and the increase in the size of metal islands are competing processes in the initial growth of metal films, and those processes depend on a number of factors, such as deposition rate, surface temperature, a number of surface defects, and parameters of adatom-surface interactions [45,48,49]. In turn, the interaction of metal atoms with a substrate surfaces is determined by the diffusion barrier $E_{d}$ and the ratio of the adsorption energy $E_{a}$ of the metal atom on the substrate to the metal cohesive energy $E_{c}$.

Several research papers provide an extensive overview of the influence of deposition rate and surface diffusion on the growth of thin films [64,65]. According to the proposed model, the island density increases as a power law of the deposition rate $n_{i}∼R^{p}$ (0<p<1). Therefore, faster deposition leads to the formation of more nucleation sites, resulting in a smaller grain size and allowing a smaller percolation thickness during film growth [50]. Increasing the deposition rate is a useful technique to grow continuous ultrathin metal films on surfaces, which are non-wettable by metal and characterized by low values of $E_{a}/E_{c}$ and $E_{d}$.

Recent research reports of Liu et al. present experimental data on the interaction of various metal adatoms with freestanding graphene [45,48]. We also used these data for the description of graphene monolayers deposited on SiO_2_/Si substrates because their weak interaction causes negligible impact on the electronic properties of graphene [66]. The ratio $E_{a}/E_{c}$ is quite low for the considered metallic adatoms on graphene and equals 0.024 and 0.090 for gold and copper, respectively, which demonstrates that adatoms interact more strongly among themselves than with graphene, and therefore, the growth of metal films on graphene can be described by the Volmer–Weber model [67]. The difference in morphology of copper and gold films can be explained by different diffusion barriers, which are 0.0037 and 0.0074 eV for copper and gold, respectively, and an almost three-fold difference in the corresponding atomic weights. Therefore, copper adatoms have higher mobility and diffuse faster on graphene compared to gold, which follows the Arrhenius equation for the hopping rate: $\upsilon =\upsilon_{0}\exp\left(-E_{d}/kT\right)$, where $\upsilon_{0}∼1/\sqrt{m}$, m is the adatom mass, k is the Boltzmann constant, and T is the absolute temperature [68,69]. Higher mobility lowers the number of secondary nucleation sites that are mainly responsible for overgrowing gaps between metallic islands. This process leads to enlargement of metallic islands and formation of wide gaps between them, which is shown in Figure 3b for 25 nm-thick copper films.

**Figure 6:**
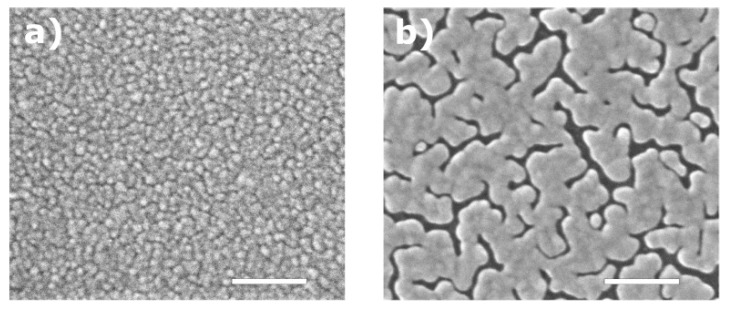
SEM micrographs (scale bar = 200 nm) of thin copper films (25 nm-thick) deposited on SiO_2_/Si substrates without (**a**) and with (**b**) graphene with a rate of 1 Å/s at 150 °C.

Higher growth temperatures increase the mobility of adatoms, which in specific cases, can lead to quasi-columnar growth. This type of growth differs from traditional Volmer–Weber growth by a steadily rising ratio of the film thickness to the filling of the substrate surface by the metal. We observed quasi-columnar growth during the deposition of copper at a rate of 1 Å/s on the graphene-covered SiO_2_/Si substrate heated at 150 °C (Figure 6). The metal filling factor for 25 nm-thick copper films deposited at 150 °C equals 84%, while the one obtained at 20 °C equals 92%. Both these films are percolated; however, the sheet resistance of the film deposited at 20 °C is 20% lower (19.0 Ohm/sq).

## 4. Conclusions

We studied the growth and properties of continuous thin films of gold and copper on monolayer graphene transferred onto silicon dioxide substrates. We presented experimental measurements for films with thicknesses of 25 and 50 nm to provide their optical, electrical, and structural properties. Table A1 and Table A2 in the Appendix A summarize the optical constants of the corresponding thin films in the wavelength range of 300–3300 nm. The results for thin gold films on graphene are not significantly different from those for gold films grown on pure thermally oxidized silicon. The variation in FOM $\left(-\varepsilon^{\prime }/\varepsilon^{\prime \prime }\right)$ obtained for these samples deposited at the rate of 1 and 5 Å/s is less than 15%. In addition, the properties of gold films show only a weak dependence on the evaporation rate. The situation is drastically different for thin copper films, whose properties strongly depend on both the type of substrate (SiO_2_/Si substrates with or without graphene) and the evaporation rate. We could not grow a continuous 25 nm-thick copper film on graphene without voids at low rates (~1 Å/s). However, higher evaporation rates (>10 Å/s) provide continuous and conductive films on graphene, which is explained by the growth kinetics of this metal. Increasing the evaporation rate from 1 to 15 Å/s improved the optical FOM and reduced the sheet resistance of copper films on graphene by 18- and 7.5-fold, respectively.

The deposition of continuous thin copper films on graphene requires high evaporation rates owing to the fast mobility of copper adatoms. Higher evaporation rates produce more initial nucleation centers, which compensates for the diffusion of copper atoms and allows the deposition of continuous films without voids. In contrast, the mobility of copper adatoms increases with increasing growth temperature. We demonstrated that substrate heating leads to quasi-columnar growth of copper on graphene, which is the limiting case of Volmer–Weber-type growth. Therefore, control of the growth temperature and evaporation rate provides an effective way to grow thin copper films with a predefined ratio of film thickness to the fraction of metal filling. In addition, due to higher adatom mobility, the formation of metal films on graphene occurs for thicknesses much higher than those on other types of substrates. This makes graphene a convenient model surface to study the kinetics of metal growth, including such processes as coalescence and percolation.

In summary, our data on the structure of thin films of copper and gold deposited on graphene as well as their optical and electrical properties will help researchers to simulate and develop various photonic, plasmonic, and optoelectronic devices. Although graphene-nanometallic structures are of high importance for practical applications, the deposition of high-optical-quality metal films on graphene still requires some technological effort. We believe that our results will make an impact in this area. In our work, we performed a comprehensive study of the properties of metal films deposited using different regimes and discussed the underlying kinetics mechanisms of their growth. In addition, the described percolated metal films with high aspect ratios can form the foundation for future devices utilizing optical resonant phenomena.

## Appendix A

**Table A1:** The measured real *ε*′ and imaginary *ε*″ parts of thin gold films on graphene as well as their refractive indices *n* and extinction coefficients *k* (at room temperature). The data at the most widely used laser wavelengths are in bold.

		*t* = 25 nm (rate = 5 A/s)				*t* = 50 nm (rate = 5 A/s)			
E (eV)	*λ* (nm)	*ε*′	*ε*″	*n*	*k*	*ε*′	*ε*″	*n*	*k*
0.38	3298	−517	94.9	2.08	22.8	−528	91.3	1.98	23.1
0.45	2739	−359	55.3	1.45	19.0	−366	53.1	1.38	19.2
0.53	2343	−262	34.9	1.08	16.2	−267	33.5	1.02	16.4
0.61	2046	−199	23.5	0.83	14.1	−203	22.5	0.79	14.3
0.68	1816	−155	16.6	0.66	12.5	−158	15.8	0.63	12.6
0.76	1633	−125	12.2	0.54	11.2	−127	11.6	0.52	11.3
**0.80**	**1550**	**−111**	**10.6**	**0.49**	**10.5**	**−113**	**10.0**	**0.47**	**10.7**
0.84	1483	−102	9.24	0.46	10.1	−104	8.82	0.43	10.2
0.91	1359	−84.0	7.23	0.39	9.17	−85.7	6.88	0.37	9.27
**0.95**	**1310**	**−77.5**	**6.53**	**0.37**	**8.81**	**−79.1**	**6.21**	**0.35**	**8.90**
0.99	1253	−70.3	5.79	0.34	8.39	−71.8	5.50	0.32	8.48
1.07	1163	−59.5	4.75	0.31	7.72	−60.8	4.50	0.29	7.80
1.14	1085	−50.7	3.98	0.28	7.13	−51.9	3.76	0.26	7.21
**1.16**	**1064**	**−48.0**	**3.76**	**0.27**	**6.93**	**−49.1**	**3.54**	**0.25**	**7.01**
1.22	1017	−43.5	3.41	0.26	6.60	−44.6	3.21	0.24	6.68
1.30	956.7	−37.6	2.98	0.24	6.13	−38.5	2.79	0.22	6.21
1.37	903.3	−32.5	2.65	0.23	5.71	−33.4	2.47	0.21	5.79
1.45	855.5	−28.3	2.41	0.23	5.32	−29.1	2.23	0.21	5.40
1.53	812.5	−24.6	2.23	0.22	4.97	−25.4	2.05	0.20	5.04
**1.59**	**780.0**	**−22.0**	**2.12**	**0.23**	**4.69**	**−22.7**	**1.94**	**0.20**	**4.77**
1.60	773.7	−21.4	2.11	0.23	4.64	−22.2	1.92	0.20	4.71
1.68	738.3	−18.7	2.03	0.23	4.33	−19.4	1.83	0.21	4.41
1.76	706.1	−16.2	1.99	0.25	4.04	−16.9	1.78	0.22	4.12
1.83	676.6	−14.1	1.98	0.26	3.76	−14.8	1.76	0.23	3.85
1.91	649.4	−12.2	2.01	0.29	3.50	−12.8	1.77	0.25	3.59
**1.96**	**633.0**	**−10.8**	**2.06**	**0.31**	**3.30**	**−11.5**	**1.76**	**0.26**	**3.39**
1.99	624.3	−10.4	2.08	0.32	3.25	−11.1	1.81	0.27	3.34
2.06	601.1	−8.87	2.18	0.36	3.00	−9.49	1.88	0.30	3.10
2.14	579.6	−7.44	2.32	0.42	2.76	−8.05	1.99	0.35	2.86
2.22	559.5	−6.14	2.52	0.50	2.53	−6.73	2.14	0.41	2.63
2.29	540.8	−4.95	2.76	0.60	2.30	−5.52	2.35	0.49	2.40
**2.33**	**532.0**	**−4.27**	**2.95**	**0.68**	**2.18**	**−4.83**	**2.50**	**0.55**	**2.27**
2.37	523.3	−3.87	3.08	0.73	2.10	−4.42	2.62	0.60	2.19
2.45	506.9	−2.90	3.48	0.90	1.93	−3.41	2.98	0.75	1.99
2.52	491.5	−2.06	3.96	1.10	1.81	−2.53	3.43	0.93	1.84
2.60	477.0	−1.39	4.53	1.29	1.75	−1.81	3.99	1.13	1.76
2.68	463.3	−0.94	5.14	1.46	1.76	−1.34	4.61	1.32	1.75
2.75	450.4	−0.71	5.73	1.59	1.80	−1.13	5.21	1.45	1.80
2.83	438.2	−0.71	6.18	1.66	1.86	−1.20	5.60	1.50	1.86
2.91	426.7	−0.82	6.42	1.68	1.91	−1.32	5.71	1.51	1.89
2.98	415.7	−0.90	6.47	1.68	1.93	−1.33	5.62	1.49	1.88
3.06	405.3	−0.87	6.43	1.68	1.92	−1.17	5.49	1.49	1.84
3.14	395.4	−0.73	6.39	1.69	1.89	−0.90	5.43	1.52	1.79
3.21	385.9	−0.53	6.43	1.72	1.87	−0.60	5.49	1.57	1.75
3.29	376.9	−0.34	6.57	1.77	1.86	−0.34	5.66	1.63	1.73
3.37	368.4	−0.20	6.80	1.82	1.87	−0.16	5.92	1.70	1.74
3.44	360.2	−0.17	7.10	1.86	1.91	−0.10	6.22	1.75	1.78
3.52	352.3	−0.30	7.42	1.89	1.97	−0.20	6.53	1.78	1.84
3.60	344.8	−0.58	7.70	1.89	2.04	−0.44	6.79	1.78	1.90
3.67	337.6	−0.97	7.90	1.87	2.11	−0.77	6.96	1.77	1.97
3.75	330.7	−1.46	7.98	1.82	2.19	−1.19	7.02	1.72	2.04
3.83	324.1	−2.01	7.85	1.75	2.25	−1.64	6.89	1.65	2.09
3.90	317.7	−2.53	7.60	1.65	2.30	−2.07	6.65	1.57	2.13
3.98	311.6	−2.99	7.19	1.55	2.32	−2.43	6.29	1.47	2.14
4.06	305.7	−3.32	6.68	1.44	2.32	−2.69	5.84	1.37	2.14
4.13	300.0	−3.59	6.13	1.33	2.31	−2.89	5.37	1.27	2.12

**Table A2:** The measured real *ε*′ and imaginary *ε*″ parts of thin copper films on graphene as well as their refractive indices *n* and extinction coefficients *k* (at room temperature). The data at the most widely used laser wavelengths are in bold.

		*t* = 25 nm (rate = 15 A/s)				*t* = 50 nm (rate = 15 A/s)			
E (eV)	*λ* (nm)	*ε*′	*ε*″	*n*	*k*	*ε*′	*ε*″	*n*	*k*
0.38	3298	−456	99.5	2.32	21.5	−502	97.3	2.16	22.5
0.45	2739	−318	58.4	1.63	17.9	−349	56.9	1.52	18.7
0.53	2343	−233	37.1	1.21	15.3	−255	36.0	1.12	16.0
0.61	2046	−177	25.1	0.94	13.4	−194	24.3	0.87	14.0
0.68	1816	−139	17.7	0.75	11.8	−152	17.2	0.70	12.3
0.76	1633	−111	13.1	0.62	10.6	−122	12.7	0.57	11.0
**0.80**	**1550**	**−99.7**	**11.3**	**0.57**	**10.0**	**−109**	**10.9**	**0.52**	**10.5**
0.84	1483	−90.8	10.0	0.52	9.54	−99.3	9.66	0.48	9.98
0.91	1359	−75.2	7.87	0.45	8.68	−82.3	7.60	0.42	9.08
**0.95**	**1310**	**−69.4**	**7.14**	**0.43**	**8.34**	**−76.0**	**6.89**	**0.39**	**8.73**
0.99	1253	−63.0	6.35	0.40	7.94	−69.0	6.13	0.37	8.31
1.07	1163	−53.3	5.26	0.36	7.31	−58.4	5.08	0.33	7.65
1.14	1085	−45.5	4.46	0.33	6.75	−49.9	4.30	0.30	7.07
**1.16**	**1064**	**−43.0**	**4.23**	**0.32**	**6.57**	**−47.3**	**4.08**	**0.30**	**6.88**
1.22	1017	−39.0	3.87	0.31	6.26	−42.9	3.73	0.28	6.56
1.30	957	−33.7	3.43	0.29	5.81	−37.1	3.31	0.27	6.10
1.37	903	−29.2	3.10	0.29	5.41	−32.2	3.00	0.26	5.68
1.45	856	−25.4	2.87	0.28	5.04	−28.1	2.77	0.26	5.31
1.53	813	−22.1	2.71	0.29	4.71	−24.5	2.61	0.26	4.96
**1.59**	**780**	**−19.7**	**2.62**	**0.29**	**4.45**	**−21.9**	**2.53**	**0.27**	**4.69**
1.60	774	−19.2	2.61	0.30	4.39	−21.4	2.52	0.27	4.64
1.68	738	−16.8	2.56	0.31	4.11	−18.8	2.47	0.28	4.34
1.76	706	−14.6	2.56	0.33	3.83	−16.4	2.48	0.31	4.06
1.83	677	−12.6	2.62	0.37	3.58	−14.3	2.52	0.33	3.80
1.91	649	−10.9	2.72	0.41	3.33	−12.4	2.62	0.37	3.55
**1.96**	**633**	**−9.71**	**2.84**	**0.45**	**3.15**	**−11.1**	**2.74**	**0.41**	**3.36**
1.99	624	−9.37	2.89	0.47	3.10	−10.8	2.78	0.42	3.31
2.06	601	−8.00	3.13	0.54	2.88	−9.26	3.00	0.49	3.08
2.14	580	−6.78	3.45	0.64	2.68	−7.92	3.31	0.58	2.87
2.22	560	−5.74	3.88	0.77	2.52	−6.76	3.71	0.69	2.69
2.29	541	−4.95	4.39	0.91	2.40	−5.81	4.21	0.83	2.55
**2.33**	**532**	**−4.60**	**4.71**	**1.00**	**2.36**	**−5.35**	**4.56**	**0.92**	**2.49**
2.37	523	−4.46	4.91	1.04	2.35	−5.14	4.79	0.97	2.47
2.45	507	−4.25	5.31	1.13	2.35	−4.80	5.33	1.09	2.45
2.52	492	−4.23	5.45	1.16	2.36	−4.74	5.66	1.15	2.46
2.60	477	−4.17	5.32	1.14	2.34	−4.79	5.67	1.15	2.47
2.68	463	−3.96	5.08	1.11	2.28	−4.73	5.44	1.11	2.44
2.75	450	−3.63	4.84	1.10	2.20	−4.50	5.13	1.08	2.38
2.83	438	−3.22	4.70	1.11	2.11	−4.14	4.87	1.06	2.29
2.91	427	−2.82	4.64	1.14	2.03	−3.73	4.70	1.07	2.21
2.98	416	−2.45	4.65	1.19	1.96	−3.32	4.62	1.09	2.12
3.06	405	−2.14	4.72	1.23	1.91	−2.96	4.61	1.12	2.05
3.14	395	−1.89	4.82	1.28	1.88	−2.65	4.65	1.16	2.00
3.21	386	−1.70	4.94	1.33	1.86	−2.40	4.71	1.20	1.96
3.29	377	−1.58	5.06	1.36	1.85	−2.22	4.79	1.24	1.94
3.37	368	−1.52	5.17	1.39	1.86	−2.09	4.87	1.27	1.92
3.44	360	−1.52	5.26	1.41	1.87	−2.02	4.93	1.29	1.92
3.52	352	−1.57	5.31	1.41	1.88	−2.00	4.96	1.29	1.92
3.60	345	−1.65	5.32	1.40	1.90	−2.02	4.97	1.29	1.92
3.67	338	−1.75	5.30	1.38	1.92	−2.06	4.94	1.28	1.93
3.75	331	−1.87	5.23	1.36	1.93	−2.12	4.88	1.26	1.93
3.83	324	−1.99	5.11	1.32	1.93	−2.18	4.76	1.24	1.93
3.90	318	−2.10	4.95	1.28	1.93	−2.24	4.63	1.20	1.92
3.98	312	−2.20	4.77	1.24	1.93	−2.29	4.46	1.17	1.91
4.06	306	−2.26	4.55	1.19	1.92	−2.32	4.27	1.13	1.90
4.13	300	−2.32	4.33	1.14	1.90	−2.34	4.07	1.08	1.88

## References

[1] Grigorenko A.N., Polini M., Novoselov K.S. (2012). Graphene plasmonics. Nat. Photonics.

[2] Li X., Zhu J., Wei B. (2016). Hybrid nanostructures of metal/two-dimensional nanomaterials for plasmon-enhanced applications. Chem. Soc. Rev..

[3] Giubileo F., Di Bartolomeo A. (2017). The role of contact resistance in graphene field-effect devices. Prog. Surf. Sci..

[4] Nair R.R., Blake P., Grigorenko A.N., Novoselov K.S., Booth T.J., Stauber T., Peres N.M.R., Geim A.K. (2008). Fine structure constant defines visual transparency of graphene. Science.

[5] Li Z.Q., Henriksen E.A., Jiang Z., Hao Z., Martin M.C., Kim P., Stormer H.L., Basov D.N. (2008). Dirac charge dynamics in graphene by infrared spectroscopy. Nat. Phys..

[6] Dawlaty J.M., Shivaraman S., Strait J., George P., Chandrashekhar M., Rana F., Spencer M.G., Veksler D., Chen Y. (2008). Measurement of the optical absorption spectra of epitaxial graphene from terahertz to visible. Appl. Phys. Lett..

[7] Geim A.K., Novoselov K.S. (2007). The rise of graphene. Nat. Mater..

[8] Bolotin K.I., Sikes K.J., Jiang Z., Klima M., Fudenberg G., Hone J., Kim P., Stormer H.L. (2008). Ultrahigh electron mobility in suspended graphene. Solid State Commun..

[9] Balandin A.A., Ghosh S., Bao W., Calizo I., Teweldebrhan D., Miao F., Lau C.N. (2008). Superior thermal conductivity of single-layer graphene. Nano Lett..

[10] Balandin A.A. (2011). Thermal properties of graphene and nanostructured carbon materials. Nat. Mater..

[11] Lee C., Wei X., Kysar J.W., Hone J. (2008). Measurement of the elastic properties and intrinsic strength of monolayer graphene. Science.

[12] Leenaerts O., Partoens B., Peeters F.M. (2008). Graphene: A perfect nanoballoon. Appl. Phys. Lett..

[13] Bunch J.S., Verbridge S.S., Alden J.S., van der Zande A.M., Parpia J.M., Craighead H.G., McEuen P.L. (2008). Impermeable atomic membranes from graphene sheets. Nano Lett..

[14] Hong J., Lee S., Lee S., Han H., Mahata C., Yeon H.-W., Koo B., Kim S.-I., Nam T., Byun K. (2014). Graphene as an atomically thin barrier to Cu diffusion into Si. Nanoscale.

[15] Kim K., Regan W., Geng B., Alemán B., Kessler B.M., Wang F., Crommie M.F., Zettl A. (2010). High-temperature stability of suspended single-layer graphene. Phys. Status Solidi RRL.

[16] Nan H.Y., Ni Z.H., Wang J., Zafar Z., Shi Z.X., Wang Y.Y. (2013). The thermal stability of graphene in air investigated by Raman spectroscopy. J. Raman Spectrosc..

[17] Stebunov Y.V., Arsenin A.V., Volkov V.S. (2018). Functionalization of chemically derived graphene for surface plasmon resonance (SPR) biosensors. Chemically Derived Graphene: Functionalization, Properties and Applications.

[18] Stebunov Y.V., Aftenieva O.A., Arsenin A.V., Volkov V.S. (2015). Highly sensitive and selective sensor chips with graphene-oxide linking layer. ACS Appl. Mater. Interfaces.

[19] Echtermeyer T.J., Britnell L., Jasnos P.K., Lombardo A., Gorbachev R.V., Grigorenko A.N., Geim A.K., Ferrari A.C., Novoselov K.S. (2011). Strong plasmonic enhancement of photovoltage in graphene. Nat. Commun..

[20] Liu Y., Cheng R., Liao L., Zhou H., Bai J., Liu G., Liu L., Huang Y., Duan X. (2011). Plasmon resonance enhanced multicolour photodetection by graphene. Nat. Commun..

[21] Fang Z., Liu Z., Wang Y., Ajayan P.M., Nordlander P., Halas N.J. (2012). Graphene-antenna sandwich photodetector. Nano Lett..

[22] Kim J., Son H., Cho D.J., Geng B., Regan W., Shi S., Kim K., Zettl A., Shen Y.-R., Wang F. (2012). Electrical control of optical plasmon resonance with graphene. Nano Lett..

[23] Sun Z., Martinez A., Wang F. (2016). Optical modulators with 2D layered materials. Nat. Photonics.

[24] Fang J., Wang D., DeVault C.T., Chung T.-F., Chen Y.P., Boltasseva A., Shalaev V.M., Kildishev A.V. (2017). Enhanced graphene photodetector with fractal metasurface. Nano Lett..

[25] Kim Y., Lee J., Yeom M.S., Shin J.W., Kim H., Cui Y., Kysar J.W., Hone J., Jung Y., Jeon S. (2013). Strengthening effect of single-atomic-layer graphene in metal–graphene nanolayered composites. Nat. Commun..

[26] Hwang B., Kim W., Kim J., Lee S., Lim S., Kim S., Oh S.H., Ryu S., Han S.M. (2017). Role of graphene in reducing fatigue damage in Cu/Gr nanolayered composite. Nano Lett..

[27] Kim M., Kang P., Leem J., Nam S. (2017). A stretchable crumpled graphene photodetector with plasmonically enhanced photoresponsivity. Nanoscale.

[28] Kholmanov I.N., Magnuson C.W., Aliev A.E., Li H., Zhang B., Suk J.W., Zhang L.L., Peng E., Mousavi S.H., Khanikaev A.B. (2012). Improved electrical conductivity of graphene films integrated with metal nanowires. Nano Lett..

[29] Kim T.-T., Kim H., Kenney M., Park H.S., Kim H.-D., Min B., Zhang S. (2018). Amplitude modulation of anomalously refracted terahertz waves with gated-graphene metasurfaces. Adv. Opt. Mater..

[30] Lu C., Hu X., Shi K., Hu Q., Zhu R., Yang H., Gong Q. (2015). An actively ultrafast tunable giant slow-light effect in ultrathin nonlinear metasurfaces. Light Sci. Appl..

[31] Aygar A.M., Balci O., Cakmakyapan S., Kocabas C., Caglayan H., Ozbay E. (2016). Comparison of back and top gating schemes with tunable graphene fractal metasurfaces. ACS Photonics.

[32] Zhao Y.T., Wu B., Huang B.J., Cheng Q. (2017). Switchable broadband terahertz absorber/reflector enabled by hybrid graphene-gold metasurface. Opt. Express.

[33] Marin B.C., Ramirez J., Root S.E., Aklile E., Lipomi D.J. (2017). Metallic nanoislands on graphene: A metamaterial for chemical, mechanical, optical, and biological applications. Nanoscale Horiz..

[34] Turcheniuk K., Boukherroub R., Szunerits S. (2015). Gold–graphene nanocomposites for sensing and biomedical applications. J. Mater. Chem. B.

[35] Leem J., Wang M.C., Kang P., Nam S. (2015). Mechanically self-assembled, three-dimensional graphene–gold hybrid nanostructures for advanced nanoplasmonic sensors. Nano Lett..

[36] Park D.-W., Schendel A.A., Mikael S., Brodnick S.K., Richner T.J., Ness J.P., Hayat M.R., Atry F., Frye S.T., Pashaie R. (2014). Graphene-based carbon-layered electrode array technology for neural imaging and optogenetic applications. Nat. Commun..

[37] Kuzum D., Takano H., Shim E., Reed J.C., Juul H., Richardson A.G., de Vries J., Bink H., Dichter M.A., Lucas T.H. (2014). Transparent, flexible, low noise graphene electrodes for simultaneous electrophysiology and neuroimaging. Nat. Commun..

[38] Kostarelos K., Vincent M., Hebert C., Garrido J.A. (2017). Graphene in the design and engineering of next-generation neural interfaces. Adv. Mater..

[39] Wong J., Jariwala D., Tagliabue G., Tat K., Davoyan A.R., Sherrott M.C., Atwater H.A. (2017). High photovoltaic quantum efficiency in Ultrathin van der Waals Heterostructures. ACS Nano.

[40] Iranzo D.A., Nanot S., Dias E.J.C., Epstein I., Peng C., Efetov D.K., Lundeberg M.B., Parret R., Osmond J., Hong J. (2018). Probing the ultimate plasmon confinement limits with a van der Waals heterostructure. Science.

[41] Parzefall M., Szabo A., Taniguchi T., Watanabe K., Luisier M., Novotny L. (2018). Light from Van der Waals quantum tunneling devices. arXiv.

[42] Tantiwanichapan K., Wang X., Durmaz H., Li Y., Swan A.K., Paiella R. (2017). Graphene terahertz plasmons: A combined transmission spectroscopy and Raman microscopy study. ACS Photonics.

[43] Johnson P.B., Christy R.W. (1972). Optical constants of the noble metals. Phys. Rev. B.

[44] Yakubovsky D.I., Arsenin A.V., Stebunov Y.V., Fedyanin D.Y., Volkov V.S. (2017). Optical constants and structural properties of thin gold films. Opt. Express.

[45] Liu X., Han Y., Evans J.W., Engstfeld A.K., Behm R.J., Tringides M.C., Hupalo M., Lin H.-Q., Huang L., Ho K.-M. (2015). Growth morphology and properties of metals on graphene. Progr. Surf. Sci..

[46] Ruffino F., Giannazzo F. (2017). A Review on metal nanoparticles nucleation and growth on/in graphene. Crystals.

[47] Hodas M., Siffalovic P., Jergel M., Pelletta M., Halahovets Y., Vegso K., Kotlar M., Majkova E. (2017). Kinetics of copper growth on graphene revealed by time-resolved small-angle X-ray scattering. Phys. Rev. B.

[48] Liu X., Wang C.Z., Hupalo M., Lu W.C., Tringides M.C., Yao Y.X., Ho K.M. (2012). Metals on graphene: Correlation between adatom adsorption behavior and growth morphology. Phys. Chem. Chem. Phys..

[49] Liu X., Wang C.Z., Hupalo M., Lin H.Q., Ho K.M., Tringides M.C. (2013). Metals on graphene: Interactions, growth morphology, and thermal stability. Crystals.

[50] Malureanu R., Lavrinenko A. (2015). Ultra-thin films for plasmonics: A technology overview. Nanotechnol. Rev..

[51] McPeak K.M., Jayanti S.V., Kress S.J.P., Meyer S., Iotti S., Rossinelli A., Norris D.J. (2015). Plasmonic films can easily be better: Rules and recipes. ACS Photonics.

[52] Fedyanin D.Y., Yakubovsky D.I., Kirtaev R.V., Volkov V.S. (2016). Ultralow-loss CMOS copper plasmonic waveguides. Nano Lett..

[53] Stebunov Y.V., Yakubovsky D.I., Fedyanin D.Y., Arsenin A.V., Volkov V.S. (2018). Superior sensitivity of copper-based plasmonic biosensors. Langmuir.

[54] Hu E.T., Cai Q.Y., Zhang R.J., Wei Y.F., Zhou W.C., Wang S.Y., Zheng Y.X., Wei W., Chen L.Y. (2016). Effective method to study the thickness-dependent dielectric functions of nanometal thin film. Opt. Lett..

[55] Zhang M.Y., Wang Z.Y., Zhang T.N., Zhang Y., Zhang R.J., Chen X., Sun Y., Zheng Y.X., Wang S.Y., Chen L.Y. (2016). Thickness-dependent free-electron relaxation time of Au thin films in near-infrared region. J. Nanophoton..

[56] Yakubovsky D.I., Fedyanin D.Y., Arsenin A.V., Volkov V.S. (2017). Optical constant of thin gold films: Structural morphology determined optical response. AIP Conf. Proc..

[57] Park J.H., Nagpal P., Oh S.-H., Norris D.J. (2012). Improved dielectric functions in metallic films obtained via template stripping. Appl. Phys. Lett..

[58] Reddy H., Guler U., Kildishev A.V., Boltasseva A., Shalaev V.M. (2016). Temperature-dependent optical properties of gold thin films. Opt. Mater. Express.

[59] Ferrari A.C., Meyer J.C., Scardaci V., Casiraghi C., Lazzeri M., Mauri F., Piscanec S., Jiang D., Novoselov K.S., Roth S. (2006). Raman spectrum of graphene and graphene layers. Phys. Rev. Lett..

[60] Zaporojtchenko V., Zekonyte J., Biswas A., Faupel F. (2003). Controlled growth of nano-size metal clusters on polymers by using VPD method. Surf. Sci..

[61] Schwartzkopf M., Hinz A., Polonskyi O., Strunskus T., Löhrer F.C., Körstgens V., Müller-Buschbaum P., Faupel F., Roth S.V. (2017). Role of sputter deposition rate in tailoring nanogranular gold structures on polymer surfaces. ACS Appl. Mater. Interfaces.

[62] Mahmoodi N., Rushdi A.I., Bowen J., Sabouri A., Anthony C.J., Mendes P.M., Preece J.A. (2017). Room temperature thermally evaporated thin Au film on Si suitable for application of thiol self-assembled monolayers in micro/nano-electro-mechanical-systems sensors. J. Vac. Sci. Technol. A.

[63] Marconi D., Colniţă A., Turcu I. (2016). The influence of deposition rate on the structure and morphology of gold/silicon(111) growth by molecular beam epitaxy. Anal. Lett..

[64] Venables J.A., Spiller G.D.T., Hanbucken M. (1984). Nucleation and growth of thin films. Rep. Prog. Phys..

[65] Müller B., Nedelmann L., Fischer B., Brune H., Kern K. (1996). Initial stages of Cu epitaxy on Ni(100): Postnucleation and a well-defined transition in critical island size. Phys. Rev. B.

[66] Fan X.F., Zheng W.T., Chihaia V., Shen Z.X., Kuo J.L. (2012). Interaction between graphene and the surface of SiO_2_. J. Phys.: Condens. Matter.

[67] Volmer M., Weber A. (1926). Nucleus formation in supersaturated systems. Z. Phys. Chem..

[68] Taylor J.B., Langmuir I. (1933). The evaporation of atoms, ions and electrons from caesium films on tungsten. Phys. Rev..

[69] Antczak G., Ehrlich G. (2010). Surface Diffusion: Metals, Metal Atoms, and Clusters.

